# Single-cell RNA-seq reveals the piperlongumine is a potential drug for ischemic stroke

**DOI:** 10.1371/journal.pone.0340725

**Published:** 2026-01-23

**Authors:** Jinwei Li, Xiaosi Zhu, Yan Zhang, Zixia Xu, Yurui Zhuang, Liqi Chen, Yan Cao, Chuan Chang, Yao Lv

**Affiliations:** 1 Department of Nursing, School of Health and Nursing, Wuxi Taihu University, Wuxi, China; 2 Departments of Neurosurgery, Huashan Hospital, Fudan University, Shanghai, China; 3 Department of Neurosurgery, the Quzhou Affiliated Hospital of Wenzhou Medical University, Quzhou People’s Hospital, Quzhou, China; Southern Illinois University Carbondale, UNITED STATES MINOR OUTLYING ISLANDS

## Abstract

Ischemic stroke is a cerebrovascular disease that can cause long-term neurological impairment, dementia, or death. It is the third most common cause of disability and the second leading cause of death worldwide. The aim of this study was to explore the underlying molecular mechanisms and potentially effective therapeutic drugs for ischemic stroke. Single-cell seq data (GSE174574) were downloaded from the Gene Expression Omnibus (GEO) database, and dimensionality reduction clustering was performed after quality control. Eighteen cell clusters were identified, which were annotated into 9 cell types according to specific marker genes. In addition, Gene Ontology (GO), Kyoto Encyclopedia of Genes and Genomes (KEGG) and Gene Set Enrichment Analysis (GSEA) enrichment analysis showed that apoptosis was significantly increased after ischemic brain injury, while p53 signaling pathway, TNF-a signaling pathway and mitogen-activated protein kinase (MAPK) signaling pathway may play an important role in ischemic stroke. Furthermore, Connectivity Map (CMap) analysis and molecular docking suggested that piperlongumine might be an effective drug for the treatment of ischemic stroke by binding to the proteins encoded by Actb and Cflar. Finally, in vitro and in vivo experiments conformed the effectiveness of piperlongumine. This study provided new ideas for the treatment of ischemic stroke.

## 1. Introduction

Cerebral ischemia can lead to brain damage due to focal or global or transient or permanent disruption of cerebral blood flow, which can lead to long-term neurological impairment, dementia, or death [1]. The cerebral ischemia in humans called an ischemic stroke is the third most frequent cause of disability and the second major cause of death all around the world [2]. A stroke can be classified as hemorrhagic or ischemic depending on the underlying neuropathology in humans [2]. Of all cases, ischemic stroke account for 85% [2]. The occlusion of the middle cerebral artery, which causes brain parenchymal damage in relevant regions followed by immune response and neuroinflammatory, is the primary cause of ischemic stroke [3,4]. Brain injury caused by ischemic stroke results from a series of complex neuropathological and neuropathophysiological events including oxidative stress, apoptosis, excitotoxicity, neuroinflammation, tau protein dysfunction and amyloid production [3–5]. The global disease research showed that the negative impact of stroke will increase with the aging trend of the world’s population, and it will become an extremely serious public health problem [6]. The transient MCAO model is a well-established representation of cerebral I/R injury, which closely simulates human ischemic stroke [7].Through the MCAO model, researchers can observe the dynamic changes of inflammatory response and apoptosis after ischemic brain injury, which is crucial for understanding the repair process after brain injury [8].

As a promising technology, single-cell RNA-seq (scRNA-seq) has been developed to explore gene expression dynamics at the level of single-cell. It has answered important questions in developmental biology, including cell trajectory analysis [9], the discovery of novel cell types [10], and cellular heterogeneity study [11], etc. A study showed that scRNA-seq revealed potential trajectory branches of monocyte/macrophage subsets, revealing precise transcriptional changes during neuroinflammation at the single-cell level [12]. Moreover, scRNA-Seq was used to identify a prominent Treg cell cluster among brain-infiltrating immune cells in the chronic stage after stroke in mice, and the results showed that Treg cell deficiency impaired the integrity of post-stroke brain white matter (WM) and delayed oligodendrocyte regeneration [13].

In this study, the dataset (GSE174574) was downloaded from Gene Expression Omnibus (GEO) database. Through quality control, dimensionality reduction clustering, cell clustering and annotation, it was found that cell apoptosis was significantly up-regulated after cerebral ischemia injury, in which p53 signaling pathway, TNF-a signaling pathway and mitogen-activated protein kinase (MAPK) signaling pathway may play an important role. In addition, Connectivity Map (CMap) analysis showed that piperlongumine might be an effective drug. The top three proteins of PPI were used for molecular docking with piperlongumine. The binding energy parameters of piperlongumine with Actb, Cflar and Jun were −6.344 kg/mol, −5.677 kg/mol and −4.219 kg/mol, respectively. Finally, the experiment confirmed that piperlongumine can reduce endothelial cell apoptosis, alleviate nerve damage, and improve functional recovery in MCAO mice.

## 2. Materials and methods

### 2.1. Download of data

We took “single cell RNA-seq or scRNA-seq” and “ischemic stroke” as keywords to retrieve in the GEO database (http://www.ncbi.nlm.nih.gov/geo/) and find ischemic stroke relevant datasets for mouse models of ischemic stroke. Single-cell transcriptomes isolated from brain tissue of middle cerebral artery occlusion (MCAO) and sham-operated mouse models were sequenced in GSE174574.

### 2.2. Single cell analysis

Seurat package was used to analyze the scRNA sequencing data [14]. The “CreateSeuratObject” function was used to convert the dataset into “Seurat object”, and then quality control was performed based on the percentage of mitochondrial genes as well as the expression and counts of sequencing genes. Subsequently, the “NormalizeData” function using the “LogNormalize” method was performed to normalize the data, and the “RunPCA” function was used to implement PCA. After entering the first 30 principal components, density clustering was performed to distinguish groupings, which was visualized by T-distributed statistical neighborhood embedding (tSNE). The “FindAllMarkers” function was used to identify marker-genes for each cluster and the “SingleR” package was carried out to annotate the cell type [15]. To show the proportion of each cell type, the “geom_bar” function of the “ggplot” package was used to draw percentile charts.

### 2.3. Difference analysis

“FindMarkers function” and Wilcoxon rank-sum test of Seurat were used to determine the differentially expressed genes (DEGs) of each cellular cluster compared to other cellular clusters (fold-change >1.5 and P < 0.05 were considered statistically significant). Subsequently, the “Pheatmap” package in R software was used for heatmap and the “ggplot2” package was used for volcano map.

### 2.4. Functional enrichment analysis

The screened DEGs were performed for Gene Ontology (GO) enrichment analysis, Kyoto Encyclopedia of Genes and Genomes (KEGG) enrichment analysis and Gene Set Enrichment Analysis (GSEA) via clusterProfiler package [16]. Then, the protein-protein interaction (PPI) network of related genes was constructed by STRING database and Cytoscape [17].

### 2.5. Drug prediction and molecular docking

CMap analysis was used to predict small molecule compounds capable of counteracting the effects of these DEGs, which could be potential targets for the treatment of ischemic stroke and its related diseases. CMap (https://portals.broadinstitute.org/cmap) is a database built using gene expression profiles of human cells treated with small molecule drugs. Through the same or opposite expression patterns, small molecule drugs, gene and the disease can be linked to each other. To find potential therapeutic drugs for ischemic stroke, the DEGs were compared and ranked by the Kolmogorov-Smirnov test after being uploaded to CMap. Subsequently, the structure of potential therapeutic drug piperlongumine was downloaded from the PubChem (https://pubchem.ncbi.nlm.nih.gov/) and then modified with Autodocktools 1.5.7 software. The top three targets derived from the PPI network were Actb, Cflar and Jun, which were downloaded from the RCSB Protein Data Bank (http://www.pdb.org/). These targets undergo a series of operations including ligand and water molecule removal, hydrogenation, amino acid optimization as well as patching. The pdbqt format was used as the saving format for files. Then the 3D chemical structures were created with ChemBioDraw 3D and their energy was minimized. The results were saved as MOL.2 format. The flexible keys of compounds imported into the Autodocktools 1.5.7 are rotatable by default and then the compounds were saved in pdbqt format to dock the ligand. After docking with Autodock Vina 1.1.2, PyMOL 3.0 was carried out to visualize the results.

### 2.6. Cell culture of bEnd.3

bEnd.3 cells were grown in DMEM supplemented with 10% fetal bovine serum (FBS; Inner Mongolia Opcel Biotedhnology Co.,Ltd. China), 100 units/mL of penicillin, and 100 μg/mL of streptomycin (MeilunBio, China). bEnd.3 cells were maintained in a humidified incubator at 37 °C with 5% CO2 and 95% air. All experiments were carried out when the density of cells was 90–100%. Confluent bEnd.3 cells were assigned to two groups: control group and piperlongumine group. Piperlongumine group treated with 5uM piperlongumine for 24 h.

### 2.7. CCK8

Initially, bEnd.3 cells (1000 per well) were seeded in a 96-well plate and cultured for 0, 24 and 48 hours. Subsequently, 10 μL of CCK-8 reagent was added to each well, followed by an additional 1.5-hour incubation at 37°C. Finally, optical density (OD) values were measured at 450 nm using a microplate reader to generate the proliferation curve.

### 2.8. Flow cytometry

Apoptosis was quantified using flow cytometry with the Annexin V-FITC Apoptosis Detection Kit (Beyotime, China; C1062S). Cells were harvested, washed with cold phosphate-buffered saline (PBS), and then resuspended in 1x binding buffer. Annexin V-FITC and propidium iodide (PI) were added to the cell suspension, and the mixture was incubated in the dark for 15 minutes at room temperature. Following incubation, the cells were analyzed on a flow cytometer to distinguish between viable cells, early apoptotic cells (Annexin V-FITC positive and PI negative), late apoptotic cells (Annexin V-FITC and PI positive), and necrotic cells (Annexin V-FITC negative and PI positive). Data acquisition and analysis were performed using FlowJo (10.8.1), and the results were expressed as the percentage of apoptotic cells relative to the total cell population.

### 2.9. Animals

The experimental animals were housed and bred in a barrier facility at the Medical Laboratory Animal Center of Fudan University Shanghai Medical College in a 12-h light/12-h dark cycle in accordance with the Code of Animal Ethics Committee of Fudan University Shanghai Medical College. Mice used in the experiment were euthanized by head and neck dislocation after intraperitoneal injection of 1.25% tribromoethanol anesthetic. The mice used in the experiments lived in an specific pathogen-free (SPF) grade barrier environment. The temperature was maintained at 20–26 degrees Celsius. In accordance with national standards, the mice were fed commercial laboratory animal feed that was sterilized by cobalt 60 irradiation. The mouse drinking water was pH 2.5–3.0 acidified. For all surgeries, anesthesia was induced in mice using 3% isoflurane and maintained with 2% isoflurane delivered through a nose cone. Every effort was made to minimize pain. All animal experiments were approved by the Animal Ethics Committee of Fudan University Shanghai Medical College (202305010Z).

### 2.10. Murine model of ischemic stroke

The MCAO model was established as previously described [18]. Animal ethics approval was obtained from Animal Ethics Committee of Fudan University Shanghai Medical College. Briefly, mice were anesthetized with 3% isoflurane and then fitted with a nose cone blowing 2% isoflurane for anaesthesia maintenance. The right common carotid artery (CCA), external carotid artery (ECA) and internal carotid artery (ICA) were isolated through a ventral midline incision. A nylon suture (Beijing Sunbio Biotech Co. Ltd., Beijing, China) was inserted from the external into the ICA until it blocked the origin of the middle cerebral artery (MCA). Sham-operated rats were manipulated in the same way, without MCAO. After surgery, mice were intraperitoneally injected with 45 mg/kg piperlongumine or vehicle solution. Each group contains at least 6 samples. At the end of the experiment, the mice were euthanized with an overdose of isoflurane. Throughout the experiments, all animals were treated with care, and every effort was made to reduce their pain.

### 2.11. Cerebral blood flow (CBF) measurement

Arterial spin labelling (ASL) was conducted to measure cerebral blood flow (CBF) 7 days post-stroke using an echo-planar imaging fluid-attenuated inversion recovery (EPI-FLAIR) sequence. CBF map of coronal slice (bregma −0.8 mm) was generated from ASL data as previously described [19]. CBF value was estimated as previously described [20]. ROI was drawn on the ipsilateral and contralateral hemispheres.

### 2.12. Neurological function tests

In all animals, the rotarod test and foot-fault tests were performed and the modified neurological severity score (mNSS) was obtained before stroke and at 7 days after stroke with or without piperlongumine treatment. The mNSS is a composite that is used to assess neurological functions based on motor, sensory, balance, and reflex measures, which are graded on a scale of 0–14 (normal score, 0; maximal deficit score, 14); higher scores imply greater neurological injury [21].

### 2.13. Immunofluorescence

For immunofluorescence staining, brain sections were thawed and washed with PBS buffer. Subsequently, they were blocked for 1 hour at room temperature in a PBS-based blocking solution containing 5% normal donkey serum and 0.5% Triton X-100 (Triton X-100). The sections were then incubated overnight at 4°C with the primary antibody against NeuN (dilution ratio 1:500, Abcam, Cat. No. ab104224). After washing, the sections were incubated with Alexa Fluor 488-conjugated secondary antibody (dilution ratio 1:400, Thermo Fisher Scientific, Cat. No. R37116) for 2 hours at room temperature in the dark. Finally, the sections were counterstained with DAPI (dilution ratio 1:1000, Yeasen, Cat. No. 36308ES20) for nuclear labeling, and images were acquired using a digital slide scanner (Model VS200, Olympus, Japan).

### 2.14. Statistical analysis

Each experimental procedure was conducted with a minimum of three replicates to ensure reliability. Data are expressed as the mean ± standard error of the mean (SEM) to reflect variability. Statistical analyses were performed using one-way ANOVA followed by Tukey’s post hoc test for multiple comparisons or Student’s t-test for pairwise comparisons, as appropriate, utilizing R software (version 4.3.0). The thresholds for statistical significance were defined as *p < 0.05, **p < 0.01, and ***p < 0.001, indicating increasing levels of significance.

## 3. Results

### 3.1. ScRNA-seq analysis

**Fig 1:**
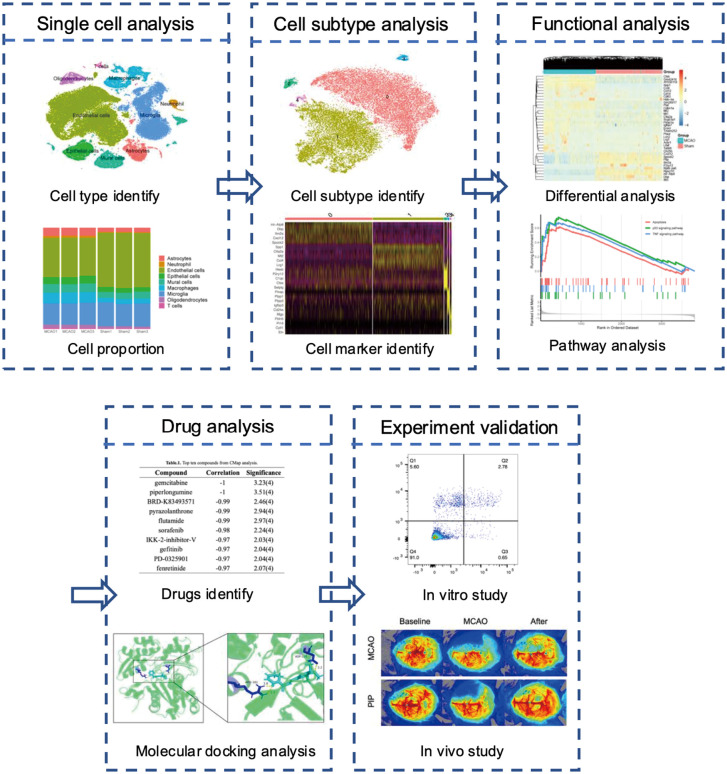
Flow chart summarizing the article.

**Fig 2:**
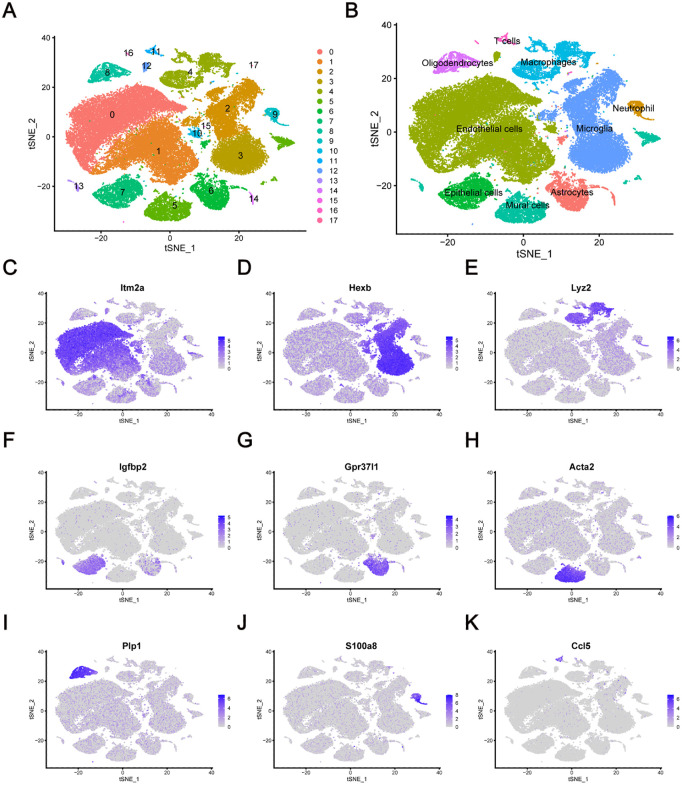
Identification of cell clusters and gene signatures during the progression of ischemic stroke progression. **(A)** Cell clusters identified and visualized by t-SNE analysis in ischemic stroke. **(B)** Differentiation of cell types visualized by t-SNE analysis in ischemic stroke. **(C-K)** Dot plots reveal the most specifically expressed marker-genes indicated in each cell cluster on the t-SNE map.

Fig 1 shows the flow of our whole study. The dataset (GSE174574) downloaded from GEO was read by R software. Subsequently, the violin plots were used to control quality. To remove the influence of mitochondrial genes and extreme genes, the number of genes sequenced was required to be kept between 200 and 3000. The cells with more than 15% mitochondrial gene expression were removed (S1A–S1C Fig). And dimensionality reduction clustering was performed after quality control. According to the analysis of the standardized variance of the average expression, 16676 genes were non-variable while 2000 genes, which had the highest average expression, were variable. Among them, S100a8, S100a9, Hbb-bs, Hba-a1, Hba-a2, Retnig, Ngp, Hbb-bt, Ccl5 and Camp were ranked in the top 10 variable genes in cell clusters (S1D Fig). Then 18 distinct cell clusters were obtained by t-SNE analysis (Fig 2A). The individual cell clusters were distinguished clearly by the expression of the top 5 marker-genes in each cluster (S1E Fig). According to the specificity of the marker-genes (Itma2a Hexb, Lyz2, Igfbp2, Gpr37I1, Acta2, Plp1, S100a8 and Ccl5) (Fig 2C–2K), 18 cell clusters were identified as 9 cell types (Endothelial cells, Microglia, Macrophages, Epithelial cells, Astrocytes, Mural cells, Oligodendrocytes, Neutrophil and T cells).

### 3.2. Identification of endothelial cell subsets

**Fig 3:**
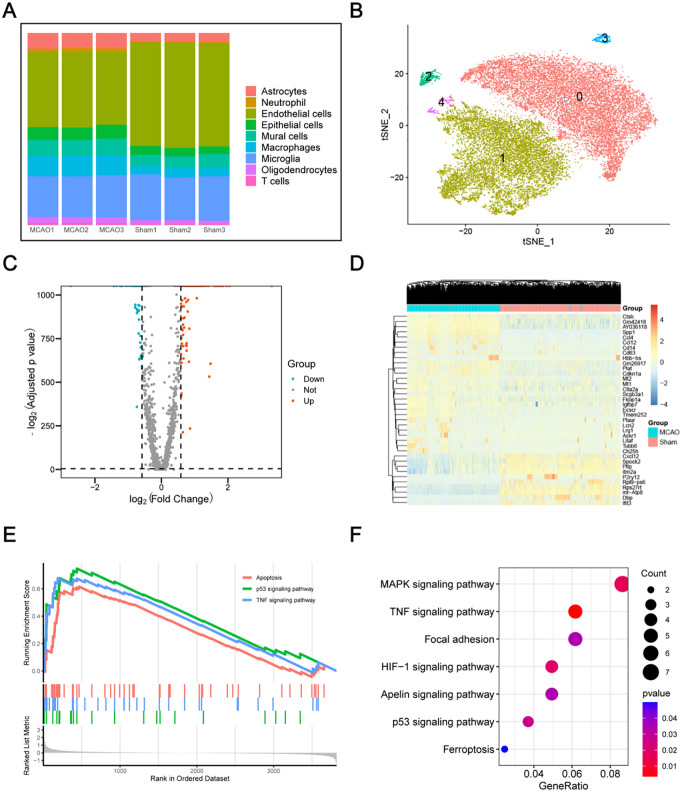
Endothelial cell subset analysis, differential analysis and functional enrichment analysis. **(A)** the proportion of each cell type in each sample. **(B)** Subsets of the endothelial cells identified and visualized by t-SNE analysis. **(C)** Volcano plot of differentially expressed genes, blue dots indicate down-regulated differentially expressed genes and red dots indicate up-regulated differentially expressed genes. **(D)** Heatmaps showing the up-regulated and down-regulated genes in each group. **(E)** GSEA analysis of differentially expressed genes. **(F)** KEGG pathway enrichment analysis of differentially expressed genes.

By analyzing the percentile chart, it was found that among the 9 cell types, endothelial cells accounted for the largest proportion (Fig 3A). Then, by further t-SNE analysis, the extracted endothelial cells could be divided into 5 subsets (Fig 3B), each of which had specific markers (S1F Fig).

### 3.3. Difference analysis and functional enrichment analysis

To explore the biological changes occurring after ischemic stroke, the endothelial cells was selected for differential analysis. Finally, 1479 differentially expressed genes (DEGs) were screened out, of which 946 genes were up-regulated while 533 genes were down-regulated (Fig 3C). Up-regulated or down-regulated genes which fold-change >4 in each group were shown in the heatmap (Fig 3D).

Then, the enrichment analysis of GO and KEGG pathway was performed. Three aspects were included in the GO enrichment analysis. In terms of biological processes (BP), DEGs were mainly enriched in “cytokine-mediated signaling pathway”, “leukocyte migration” and “rugulation of angiogenesis” (S2A Fig). The molecular function (MF) revealed that three most significantly enriched categories were “collagen-containing extracellular matrix”, “apical part of cell” and “membrane raft” (S2B Fig). The cell components (CC) showed that “cytokine receptor binding”, “G protein-coupled receptor binding” and “cytokine activity” were the top three enriched genes (S2C Fig). Meanwhile, KEGG pathway enrichment analysis showed that these DEGs were primarily enriched in the “MAPK signaling pathway” and “TNF signaling pathway” (Fig 3F). To further explore the potential mechanism of ischemic stroke, gene set enrichment analysis (GSEA) results revealed that the “apoptosis”, “p53 signaling pathway” and “TNF signaling pathway” were up-regulated after ischemic stroke (Fig 3E).

We found that apoptosis of endothelial cells was the significant mechanism of ischemic stroke. Therefore, apoptosis-related DEGs were selected for further analysis. The results of differential analysis of apoptosis-related genes were shown in S1 Table. And the PPI network, which was related to the apoptosis-related genes, showed that Actb, Jun, Cflar, Fas, Pmaipp1, Ctsl, Ctsd, Ctsb, Bax and Tnfrsf1a, were the top 10 hub genes (S2D Fig).

### 3.4. The results of drug prediction and analysis of docking

**Fig 4:**
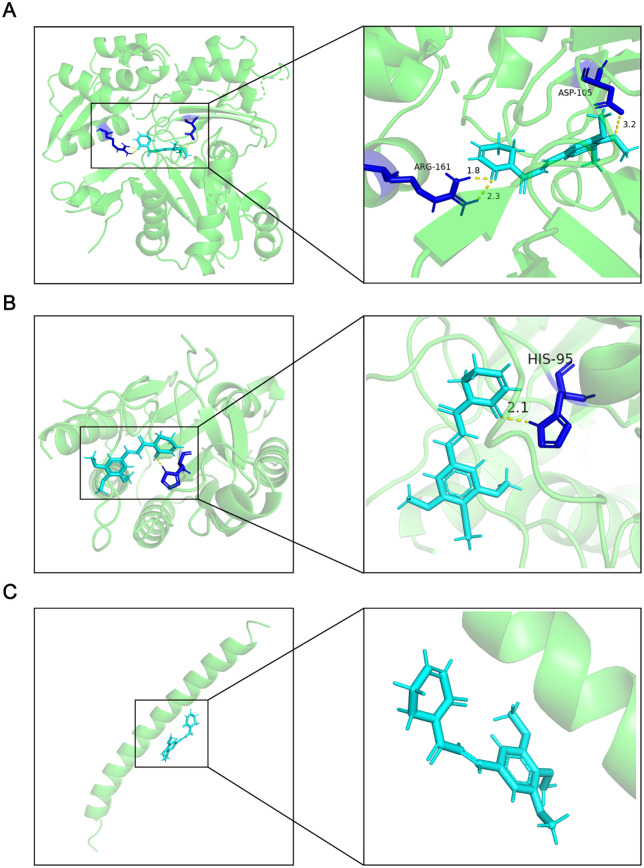
Results of molecular docking of piperlongumine with proteins encoded by Actb, Cflar and Jun. **(A)** Molecular docking results of stable binding between piperlongumine and Actb encoded protein. **(B)** Molecular docking results of stable binding between piperlongumine and Cflar encoded protein. **(C)** Molecular docking result showing that piperlongumine could not stably bind to Jun encoded protein.

The CMap database was used to predict potentially effective drugs. According to the prediction results, gemcitabine and piperlongumine were ranked equally first (Table 1). It found that the latter may have therapeutic effect through further literature searching. Subsequently, the top three genes (Actb, Jun, Cflar) in PPI were selected as receptors and piperlongumine was thought to be the ligand for molecular docking. The binding energy parameters of piperlongumine with Actb, Cflar and Jun were −6.344 kg/mol, −5.677 kg/mol and −4.219 kg/mol, respectively (Fig 4). It is generally believed that when the binding energy is less than -5kJ/mol, the ligand and the receptor can bind stably. Therefore, piperlongumine could stably bind to Actb at specific sites ARG-161 and ASP-105 through hydrogen bonding (Fig 4A). ln addition, piperlongumine could stably bind with Cflar to the specific site HIS-95 as well (Fig 4B).

**Table 1:** Top ten compounds from CMap analysis.

Compound	Correlation	Significance
Gemcitabine	−1	3.23 (4)
Piperlongumine	−1	3.51 (4)
BRD-K83493571	−0.99	2.46 (4)
Pyrazolanthrone	−0.99	2.94 (4)
Flutamide	−0.99	2.97 (4)
Sorafenib	−0.98	2.24 (4)
IKK-2-inhibitor-V	−0.97	2.03 (4)
Gefitinib	−0.97	2.04 (4)
PD-0325901	−0.97	2.04 (4)
Fenretinide	−0.97	2.07 (4)

### 3.5. Anti-apoptosis effect of piperlongumine in endothelial cells

**Fig 5:**
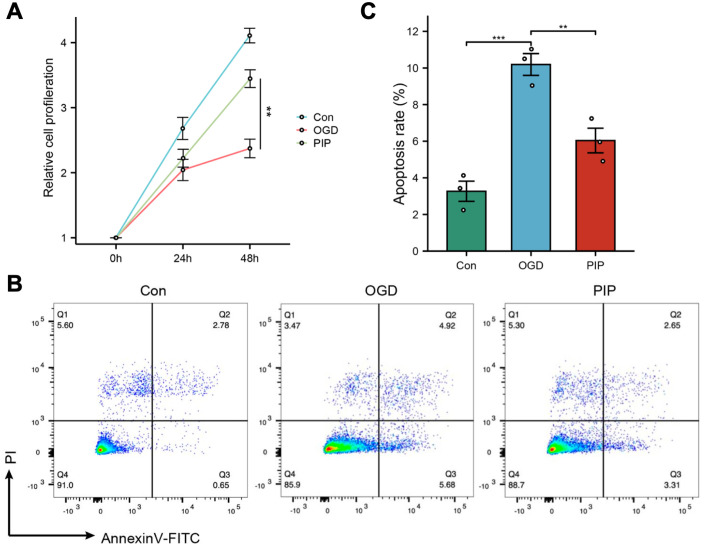
Piperlongumine inhibited apoptosis of endothelial cells. **(A)** The results of CCK8. **(B)** Representative flow cytometry dot plots. **(C)** Percentage of apoptosis rate. Abbreviation: Con, control; OGD, oxygen glucose deprivation; PIP, piperlongumine. Each experiment was repeated 3 times. Data are presented as the mean ± SEM. **p < 0.01, ***p < 0.001.

We used a Cell Counting Kit (CCK8) to determine the effect of piperlongumine on the viabilities of ischemic-injured bEnd.3 cells. As shown in Fig 5A, exposure to piperlongumine for different periods of time, the decrease in vitalities lessened compared with the control group. Besides, cell proliferation increased over time in all groups, but at 48 hours, the proliferation in the piperlongumine treatment group was significantly higher than in the OGD group. From the Fig 5B, 5C, it can be seen that the apoptosis rate is the highest in the oxygen-glucose deprivation group (OGD), significantly higher than in the control group. The expression level in the PIP treatment group is lower than OGD group, indicating that piperlongumine could inhibit the apoptosis of bEnd.3 cells.

### 3.6. Piperlongumine could improve functional recovery after ischemic stroke

**Fig 6:**
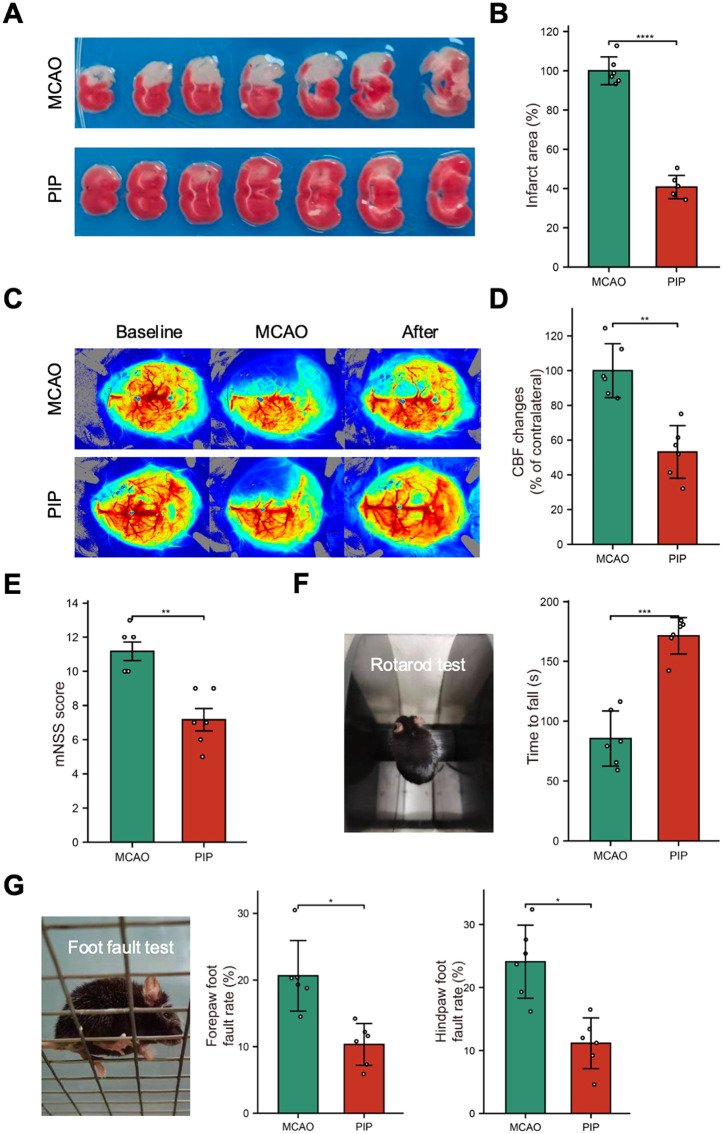
Piperlongumine improved functional recovery after ischemic stroke. **(A)** Brain sections comparison showing the infarct area before and after treatment in the MCAO model. **(B)** Percentage of intact brain region. **(C)** The tracer image demonstrates a focal reduction in cerebral blood flow, mainly involving the MCA cortical perfusion territory. **(D)** Percentage comparison of changes in cerebral blood flow. **(E)** mNSS score chart illustrating neurological deficits. **(F)** Rotarod test showing motor coordination and balance ability of mice after treatment. **(G)** Foot fault test evaluating motor precision of mice, showing the error rates of forelimbs and hindlimbs. Abbreviation: MCAO, middle cerebral artery occlusion; PIP, piperlongumine. Each group contains at least 6 samples. Data are presented as the mean ± SEM. *p < 0.05, **p < 0.01, ***p < 0.001, ****p < 0.0001**.**

The effect of piperlongumine in promoting neurological recovery was evaluated. The TTC-staining showed that piperlongumine could reduce the region of injury area (Fig 6A, 6B). The primary objective was to monitor regional cerebral blood flow (rCBF) using laser speckle imaging, with a focus on the cortical perfusion territory of the MCA. The results show that the PIP group exhibits improved cerebral blood flow post-treatment, with significant enhancements compared to the MCAO group (Fig 6C, 6D). Moreover, neurological function scores (mNSS) are better in the PIP group, suggesting reduced neural damage (Fig 6E). And, PIP treatment significantly increases the time that mice can maintain balance on the apparatus as shown in Fig 6F. Moreover, the Pip treatment group demonstrates a reduced error rate in both forelimbs and hindlimbs, indicating improved motor function as illustrated in Fig 6G. Finally, we performed the immunofluorescence staining of NeuN, a marker of neurons in nervous system. The results also show that Pip treatment group could effectively reduce neuronal loss (S3 Fig). These results validated the recovery-promoting function of piperlongumine.

## 4. Discussion

Ischemic stroke would cause severe neurological and mental damage, and even lead to dementia or death from various causes. With the development of single-cell sequencing technology, single-cell analysis had been used to reveal the transcriptional profile of ischemic stroke, but the underlying molecular mechanism and potential drugs of ischemic stroke remained to be investigated [12]. Single-cell analysis was performed after downloading the data from the GEO database to find enriched pathways and potential effective drugs. These analyses help us to further explore the potential molecular mechanism of ischemic stroke and new effective therapeutic drugs. Although the MCAO model has certain limitations in simulating clinical cerebral ischemic injury, its contribution to revealing drug action mechanisms and potential treatment strategies cannot be ignored. Through further clinical research, the clinical applicability of these findings can be better verified, thus providing more effective intervention measures for the treatment of cerebral ischemic injury [22,23].

The progression and outcome of ischemic stroke were regulated by a variety of cell types, which played different roles in ischemic stroke. It had been suggested that extracellular vesicles produced by mesenchymal stem cells and brain endothelial cells have the effect of upregulating the expression of ZO-1 and Claudin-5, as well as reducing the volume of cerebral infarction and permeability of the blood-brain barrier in rats after 24 hours of MCAO treatment [24]. As key regulators, microglia play a role in neuroprotection and functional recovery of cerebral ischemia [25]. Studies have shown that microglia, invading macrophages, invading neutrophils, T cells (e.g., γδ T cells, CD8 + cytotoxic T cells, CD4 T cells and Treg cells) gradually migrate to the injured brain region after ischemic stroke, and these cells enhance or attenuate ischemic brain parenchymal damage by inducing sterile inflammation [26]. Linhao You et al. revealed that ferroportin 1 (FPN1) of brain microvascular endothelial cells (BMVECs) is the pathway for iron to cross the blood-brain barrier, and it is controlled by hepcidin secreted by astrocytes and direct physical contact with it [27]. In addition, Endothelial S1P1 signaling may resist injury in ischemic stroke by maintaining regional blood supply and protecting brain-blood barrier [28]. Microglia, as immune cells in the central nervous system, are activated and trigger neuroinflammatory responses after ischemic brain injury. Studies have shown that by regulating the activation state of microglia, it is possible to effectively reduce the volume of cerebral infarction and improve neurological function [29,30]. Thus, the annotation of multiple cell clusters by scRNA-seq analysis would be significant for further exploration of the underlying pathogenesis and progression of ischemic stroke.

Apoptosis of endothelial cells may exacerbate ischemic stroke injury and have adverse effects on recovery. Tightly-joined endothelial cells form a transport barrier between the cerebral capillaries and brain, so apoptosis of the endothelial cells disrupts the homeostasis of the environment around the nerve cells. It has been reported that the destruction of the blood-brain barrier causes plasma to leak out of the blood vessels into the extravascular space of brain tissue, resulting in vasogenic edema [31,32]. Additionally, researches have shown that MCAO-induced apoptosis of cerebral microvascular endothelial cells or loss of endothelial cell-derived R-spondin 3 (RSPO3) can inhibit Erk activation in ischemic penumbra brain tissue and increase cerebral ischemic injury of mice [33]. PPI network analysis showed that Actb, Jun and Cflar were the most important Hub genes. It has been shown that by forming filaments into cytoskeleton, Actb protein plays a vital role in cellular processes like cell migration, cell division, and regulation of gene expression, maintaining the viabilities of cells [34,35]. Activated c-Jun N-terminal kinase (JNK) binds to the amino-terminal regions of the transcription factors ATF2 and c-Jun, resulting in phosphorylation of the active region of the transcription factor, mediating apoptosis through JNK signaling pathway [36]. Zhang et al. reported that activated Jun enhances cell survival and reproduction by binding to the promoters of key effectors of the unfolded protein response (UPR) and activating their transcription [37]. CFLAR (CASP8 and FADD-like apoptosis regulator) long isoform (CFLARL) inhibits autophagy, necroptosis, and apoptosis of T lymphocytes by inhibiting the activation of CASP8 [38]. All these provide evidence that Actb, Jun and Cflar play important roles in apoptosis.

At the end of this study, we used CMap analysis to predict the drugs used for effective treatment of ischemic stroke. After combining the analysis results and reviewing the literature, we decided to use piperlongumine as the drug for further analysis. The natural alkaloid compound piperlongumine is derived from long pepper (Piper longum) [39]. It is an anti-inflammatory, atherosclerosis-preventative, and antitumor compound [40]. In summary, piperlongumin may play a role in anti-apoptosis through certain mechanisms. Through molecular docking, it was found that piperlongumine could stably bind to Actb and Cflar. Therefore, we hypothesized that piperlongumine would interact with Actb and Cflar to mediate anti-apoptosis during the progression of ischemic stroke. And piperlongumine may be a potential therapeutic agent for ischemic stroke.

However, there were certain limitations in this study. This study explored the potential therapeutic effect of piperlongumine on MCAO through bioinformatics analysis. Although preliminary in vitro and in vivo experiments have confirmed its effectiveness, further clinical trials are needed to clarify its clinical significance. Secondly, the analysis revealed that the p53 signaling pathway, TNF-α signaling pathway, and MAPK signaling pathway are the main molecular mechanisms through which the drug exerts its effect. Subsequent rescue experiments are still needed to elucidate its molecular mechanisms.

## 5. Conclusion

In summary, this study found that piperlongumine has the function of anti-ischemic stroke injury, and proposed for the first time that piperlongumine can exert anti-cerebral microvascular endothelial cell apoptosis by binding to proteins encoded by Actb and Cflar genes, thereby reducing ischemic stroke injury and promoting recovery. Piperlongumine has a positive effect on neurological function of MCAO mice and may be a candidate for treatment of ischemic stroke.

## Supporting information

S1 Fig Quality control of single cell analysis, differentially expressed genes and specific genes for cell clusters and endothelial cell subsets. (A) The violin plots revealing the number of reads in sequencing and the expression of mitochondrial genes. (B) The ratio of the number of genes detected to the amount of RNA. (C) The violin plots revealing the number of reads in sequencing and the expression of mitochondrial genes. (D) The top 10 standardized variant genes. (E)Heatmap showing the scaled expression of the top 5 marker-genes for each of the nine cell clusters annotated. (F)Heatmap showing the scaled expression of the top 5 marker-genes for each of the subset of endothelial cells.
(DOCX)

S2 Fig GO enrichment analysis and PPI interaction network. (A) GO biological process (GO-BP) analysis of differentially expressed genes. (B) GO molecular function (GO-MF) analysis of differentially expressed genes. (C) GO cellular component (GO-CC) analysis of differentially expressed genes. (D) PPI network of proteins associated with apoptosis related genes.
(DOCX)

S3 Fig Neuroprotective effects of Piperlongumine. (A) Representative immunofluorescence staining images of brain in different groups. Green signal represents NeuN and blue signal represents DAPI. Scale bars, 100 μm. (B) Quantification of the NeuN fluorescence area. Abbreviation: MCAO, middle cerebral artery occlusion; PIP, piperlongumine. Data are presented as the mean ± SEM. **p < 0.01.
(DOCX)

S1 Table The result of differential analysis of apoptosis related genes. (DOCX)
