# Supplementary material for: Single-cell RNA-seq reveals the piperlongumine is a potential drug for ischemic stroke
Source: PLoS One. 2026 Jan 23;21(1):e0340725. doi: 10.1371/journal.pone.0340725 (PMC12829879; doi:10.1371/journal.pone.0340725)
Supplement: S1 Fig — (A) The violin plots revealing the number of reads in sequencing and the expression of mitochondrial genes. (B) The ratio of the number of genes detected to the amount of RNA. (C) The violin plots revealing the number of reads in sequencing and the expression of mitochondrial genes. (D) The top 10 standardized variant genes. (E)Heatmap showing the scaled expression of the top 5 marker-genes for each of the nine cell clusters annotated. (F)Heatmap showing the scaled expression of the top 5 marker-genes for each of the subset of endothelial cells. (DOCX) [file pone.0340725.s001.docx]

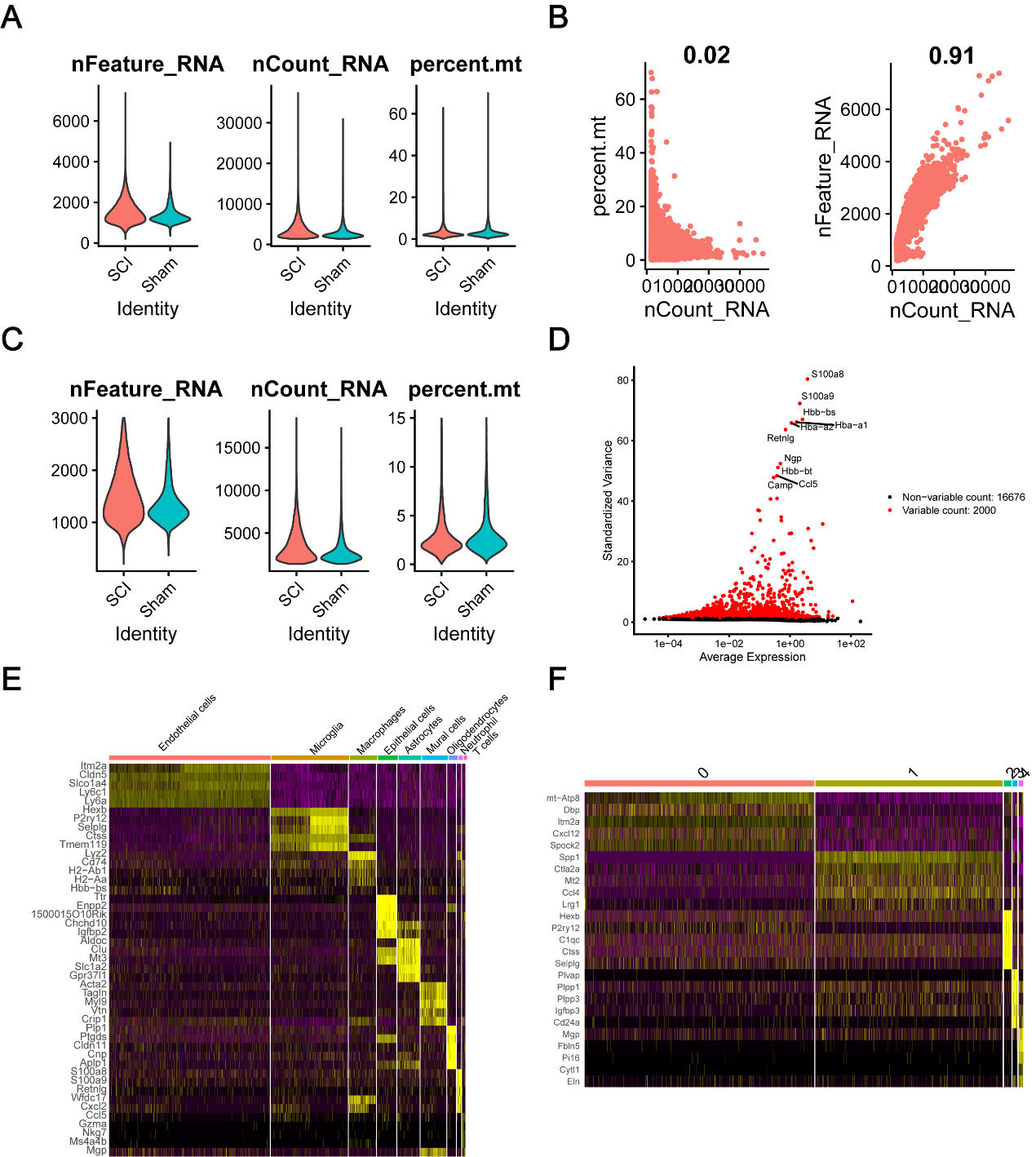


**Supplementary Figure. 1.** Quality control of single cell analysis, differentially expressed genes and specific genes for cell clusters and endothelial cell subsets. (A) The violin plots revealing the number of reads in sequencing and the expression of mitochondrial genes. (B) The ratio of the number of genes detected to the amount of RNA. (C) The violin plots revealing the number of reads in sequencing and the expression of mitochondrial genes. (D) The top 10 standardized variant genes. (E)Heatmap showing the scaled expression of the top 5 marker-genes for each of the nine cell clusters annotated. (F)Heatmap showing the scaled expression of the top 5 marker-genes for each of the subset of endothelial cells.
