# Supplementary material for: Single-cell RNA-seq reveals the piperlongumine is a potential drug for ischemic stroke
Source: PLoS One. 2026 Jan 23;21(1):e0340725. doi: 10.1371/journal.pone.0340725 (PMC12829879; doi:10.1371/journal.pone.0340725)
Supplement: S2 Fig — (A) GO biological process (GO-BP) analysis of differentially expressed genes. (B) GO molecular function (GO-MF) analysis of differentially expressed genes. (C) GO cellular component (GO-CC) analysis of differentially expressed genes. (D) PPI network of proteins associated with apoptosis related genes. (DOCX) [file pone.0340725.s002.docx]

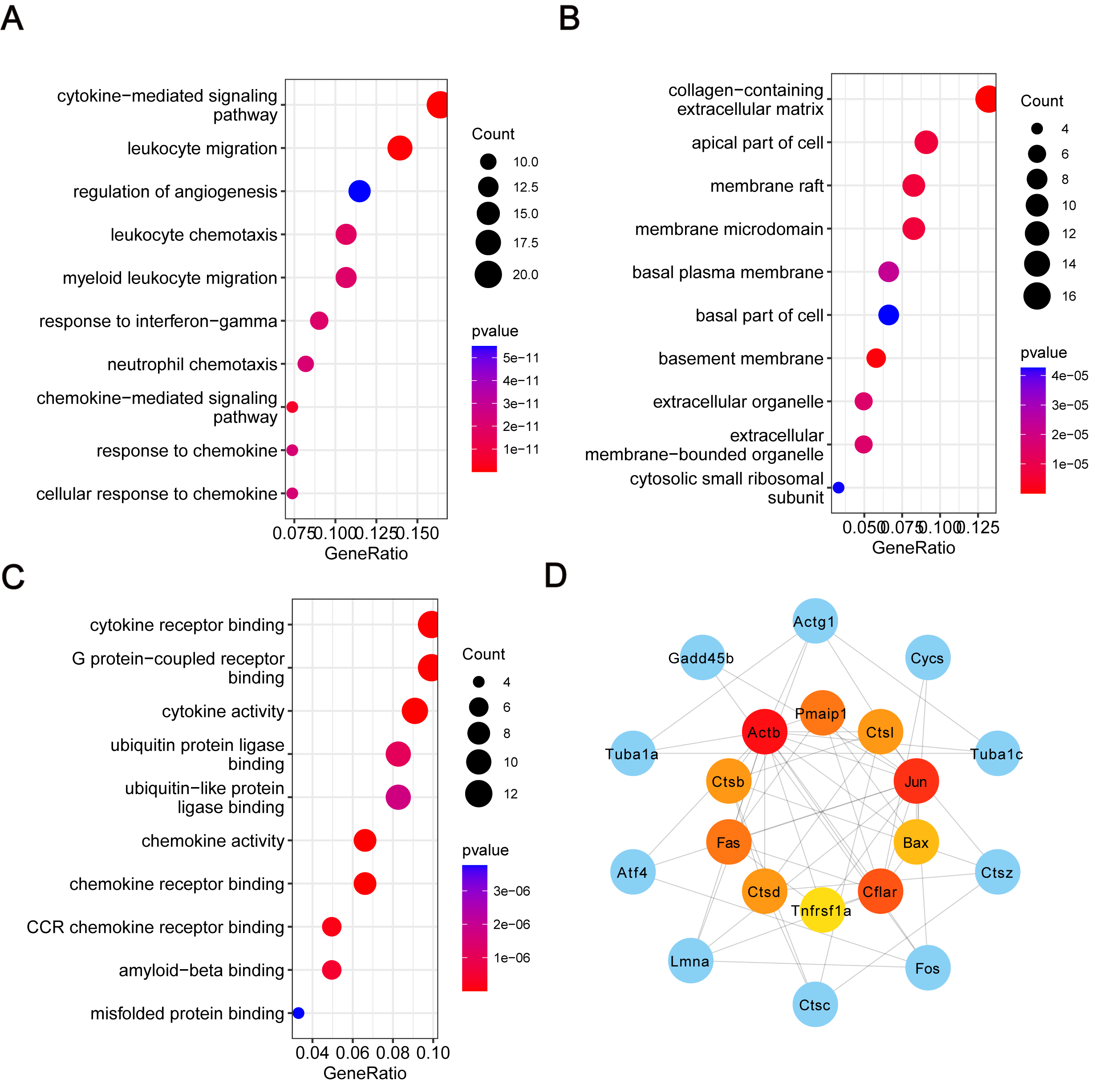


**Supplementary Figure.2.** GO enrichment analysis and PPI interaction network. (A) GO biological process (GO-BP) analysis of differentially expressed genes. (B) GO molecular function (GO-MF) analysis of differentially expressed genes. (C) GO cellular component (GO-CC) analysis of differentially expressed genes. (D) PPI network of proteins associated with apoptosis related genes.
