# Supplementary material for: Single-cell RNA-seq reveals the piperlongumine is a potential drug for ischemic stroke
Source: PLoS One. 2026 Jan 23;21(1):e0340725. doi: 10.1371/journal.pone.0340725 (PMC12829879; doi:10.1371/journal.pone.0340725)
Supplement: S3 Fig — (A) Representative immunofluorescence staining images of brain in different groups. Green signal represents NeuN and blue signal represents DAPI. Scale bars, 100 μm. (B) Quantification of the NeuN fluorescence area. Abbreviation: MCAO, middle cerebral artery occlusion; PIP, piperlongumine. Data are presented as the mean ± SEM. **p < 0.01. (DOCX) [file pone.0340725.s003.docx]

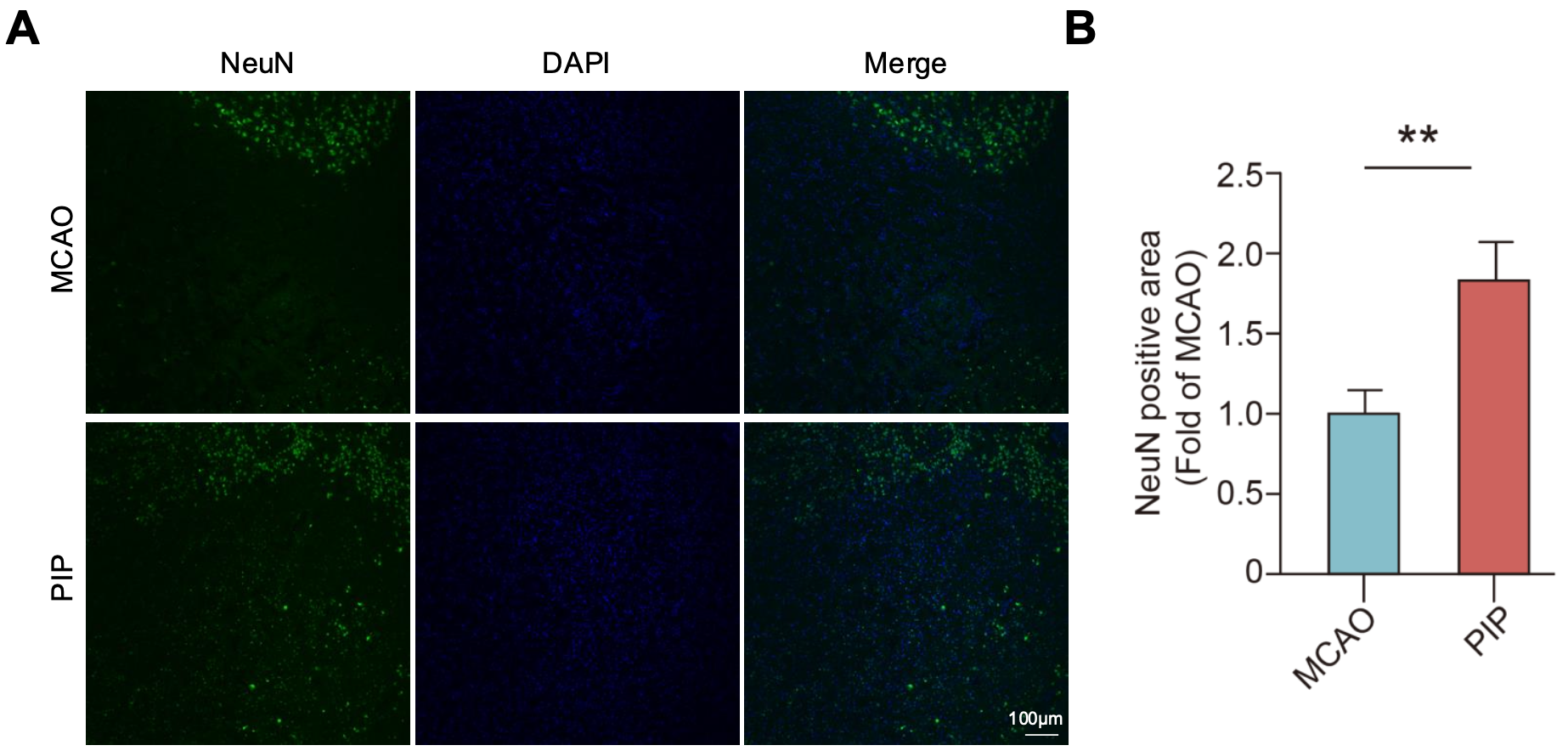


**Supplementary Figure.3.** Neuroprotective effects of Piperlongumine. (A) Representative immunofluorescence staining images of brain in different groups. Green signal represents NeuN and blue signal represents DAPI. Scale bars, 100 μm. (B) Quantification of the NeuN fluorescence area. Abbreviation: MCAO, middle cerebral artery occlusion; PIP, piperlongumine. Data are presented as the mean ± SEM. **p < 0.01.
