# Supplementary material for: Single-cell RNA-seq reveals the piperlongumine is a potential drug for ischemic stroke
Source: PLoS One. 2026 Jan 23;21(1):e0340725. doi: 10.1371/journal.pone.0340725 (PMC12829879; doi:10.1371/journal.pone.0340725)
Supplement: S1 Table — (DOCX) [file pone.0340725.s004.docx]

**Supplementary Table 1.** The result of differential analysis of apoptosis related genes.

|  | **p_val** | **avg_log2FC** | **pct.1** | **pct.2** | **p_val_adj** |
| --- | --- | --- | --- | --- | --- |
| Ctsb | 0 | 1.053374 | 0.827 | 0.495 | 0 |
| Gadd45g | 2.06E-297 | 0.802002 | 0.413 | 0.207 | 3.85E-293 |
| Ctsz | 5.60E-218 | 0.496518 | 0.535 | 0.335 | 1.05E-213 |
| Fas | 2.26E-144 | 0.454658 | 0.578 | 0.426 | 4.22E-140 |
| Fos | 1.98E-163 | 0.445603 | 0.825 | 0.675 | 3.70E-159 |
| Tnfrsf1a | 9.58E-133 | 0.412121 | 0.256 | 0.135 | 1.79E-128 |
| Lmna | 2.96E-113 | 0.401808 | 0.292 | 0.171 | 5.53E-109 |
| Tuba1c | 2.65E-189 | 0.395911 | 0.116 | 0.026 | 4.95E-185 |
| Actg1 | 3.93E-102 | 0.361843 | 0.923 | 0.904 | 7.33E-98 |
| Pmaip1 | 2.69E-69 | 0.358671 | 0.118 | 0.057 | 5.02E-65 |
| Atf4 | 3.73E-93 | 0.341347 | 0.282 | 0.173 | 6.98E-89 |
| Tuba1a | 1.98E-91 | 0.336169 | 0.747 | 0.665 | 3.70E-87 |
| Actb | 1.81E-94 | 0.335096 | 0.989 | 0.976 | 3.38E-90 |
| Ctsl | 3.30E-92 | 0.329256 | 0.568 | 0.429 | 6.16E-88 |
| Cycs | 1.14E-83 | 0.32083 | 0.545 | 0.43 | 2.13E-79 |
| Gadd45b | 1.57E-85 | 0.314238 | 0.148 | 0.072 | 2.93E-81 |
| Eif2s1 | 2.14E-67 | 0.267098 | 0.243 | 0.153 | 4.00E-63 |
| Cflar | 1.18E-42 | 0.201286 | 0.177 | 0.115 | 2.21E-38 |
| Ctsc | 1.82E-36 | 0.200761 | 0.329 | 0.251 | 3.41E-32 |
| Jun | 2.25E-14 | 0.197742 | 0.956 | 0.958 | 4.21E-10 |
| Bax | 9.94E-28 | 0.173101 | 0.47 | 0.397 | 1.86E-23 |
| Ctsd | 9.58E-102 | 0.165657 | 0.81 | 0.696 | 1.79E-97 |
